# Rwandan young people's perceptions on sexuality and relationships: Results from a qualitative study using the ‘mailbox technique’

**DOI:** 10.1080/17290376.2014.927950

**Published:** 2014-06-20

**Authors:** Kristien Michielsen, Pieter Remes, John Rugabo, Ronan Van Rossem, Marleen Temmerman

**Affiliations:** ^a^ MA in Social and Political Sciences, PhD in Social Medical Sciences, is Post-Doctoral Assistant at the International Centre for Reproductive Health, Ghent University, Ghent, Belgium.; ^b^ MA in Anthropology, PhD in Anthropology, is Radio Executive Producer and Trainer at the Development Media International, Ouagadougou, Burkina Faso; ^c^ MSc in Public Health, is coordinator of the health programme of the Rwandan Red Cross Association, Muhanga, Kamonyi, Ruhango and Bugesera (2003–2011), Rwanda; ^d^ MA in Sociology, PhD in Sociology, is Associate Professor at the Department of Sociology, Ghent University, Ghent, Belgium; ^e^ MD, PhD in Obstetrics and Gynaecology, is Full Professor of Obstetrics-Gynaecology at Ghent University, Ghent, Belgium

**Keywords:** mailbox technique, vulnerability, young people, sexuality and reproductive health, Rwanda, technique de boîte aux lettres, vulnérabilité, adolescents, santé sexuelle et reproductive, Rwanda

## Abstract

This study aimed to gain more insight into young Rwandans’ perceptions on sex and relationships, which is essential for formulating effective sexual and reproductive health (SRH) promotion interventions. Using a ‘mailbox technique’, this paper studies the spontaneous thoughts of Rwandan young people on sexuality. Mailboxes were installed in five secondary schools in the Bugesera district and students were invited to write about their ideas, secrets, wishes, desires and fears on sexuality and relationships. Of the 186 letters collected, 154 addressed SRH topics. The letters were analysed in NVivo 9 using a theoretical model on vulnerability. Two stereotypical sexual interactions co-exist: experimental sex, taking place unprepared, driven by desire among young people of the same age, and transactional sex, occurring after negotiation between older men/women and younger girls/boys in exchange for money or goods. Both types expose young people to poor, though different, SRH outcomes. Young people have little capacity to manage their vulnerability in these relationships: they have limited knowledge on SRH topics, lack adult guidance or support and have difficult access to condoms. They apply seemingly contradictory norms and behaviours concerning sexuality. In conclusion, we have formulated several recommendations for SRH interventions.

## Résumé

Cette étude vise à étudier les perceptions des jeunes Rwandais sur la sexualité et les relations, ce qui est essentiel pour la formulation des interventions efficaces en promotion de la santé sexuelle et reproductive (SSR). En utilisant une «téchnique de boîte aux lettres », cet article étudie les pensées spontanées de jeunes Rwandais. Des boîtes aux lettres ont été installées dans cinq écoles secondaires dans le district de Bugesera et les étudiants ont été invités à noter leurs idées, secrets, souhaits, désirs et craintes sur le sujet de la sexualité et les relations. Sur les 186 lettres collectées, 154 traitaient la SSR. Les lettres ont été analysées dans NVivo 9 à l'aide d'un modèle théorique de vulnérabilité. Deux interactions sexuelles stéréotypées coexistent: le sexe expérimental, qui aura lieu à l'imprévu, entrainé par le désir, entre les jeunes du même âge, et le sexe transactionnel, survenant après négociation entre hommes/femmes plus agé(e)s et entre de jeunes filles/garçons en échange d'argent ou de biens. Les deux types exposent les jeunes, de manière différente, à une mauvaise SSR. Les jeunes ont une capacité limitée de gérer leur vulnérabilité: ils ont des connaissances limitées sur des sujets de SSR, n'ont pas l'orientation ou le soutien des adultes, et ont un accès difficile aux préservatifs. Ils appliquent les normes et les comportements apparemment contradictoires concernant la sexualité. En conclusion, nous avons formulé plusieurs recommandations pour les interventions de SSR.

## Introduction

Promoting sexual and reproductive health (SRH) among young people is essential, not only for their individual benefit, but also to reverse the HIV epidemic and for development more generally (World Health Organization 2011). In sub-Saharan Africa, young people aged 15–24 remain at the centre of the HIV epidemic. Between a third and half of the new HIV infections worldwide occur in this group, resulting in a regional prevalence of 3.3% in young women and 1.4% in young men (UNAIDS 2011). The region also has the highest rate of teenage pregnancies in the world – 143 per 1000 girls aged 15–19 (Treffers 2003). Each year 2.5 million adolescent girls (10–19 years) undergo an unsafe abortion (World Health Organization 2008), making it the leading cause of death among adolescents in many countries of sub-Saharan Africa (UNFPA 2010). Overall, the consequences of pregnancy and childbirth are particularly dangerous for young girls: while 11% of all births are among adolescents, they carry 23% of the disease burden (World Health Organization 2008).

Many efforts are made to influence young people to adopt safe sexual practices and to promote SRH. However, recent literature reviews and meta-analyses found that HIV prevention and SRH promotion interventions for young people in sub-Saharan Africa only incur small changes in reported sexual behaviour (Gallant & Maticka-Tyndale 2004; Harrison, Newell, Imrie & Hoddinott 2010; Medley, Kennedy, O'Reilly & Sweat 2009; Michielsen, Chersich, Luchters, De Koker, Van Rossem & Temmerman 2010; Paul-Ebhohimhen, Poobalan & van Teijlingen 2008). Reasons for this limited success rate are not unequivocal. Implementation difficulties such as refusal or opposition of teachers to talk about condoms, resource constraints and non-adherence to project design are widespread. But also well-developed, implemented and evaluated interventions show limited effectiveness (Jewkes *et al*. 2006; Ross, Changalucha, Obasi, Todd, Plummer, Cleophas-Mazige, *et al.* 2007). Hence, it is possible that interventions do not adequately understand and address the specific vulnerabilities of young people for poor SRH, using an individual focus and failing to address social, cultural and economic, structural factors influencing sexual behaviour.

Since sexuality and sexual relationships are inherently embedded in a social context, a thorough understanding of young peoples' perceptions on sex and relationships is essential for formulating effective SRH promotion interventions. However, few studies on sexuality of young people in Africa go beyond describing HIV risk-related behaviours. Harrison (2008) studied how young South Africans construct their sexuality. She concluded that sexuality is stigmatized, especially for young women, and that a dichotomy of love and romance versus stigma and secrecy frames the sexuality discourse of young people. Maticka-Tyndale, Gallant, Brouillard-Coyle, Holland, Metcalfe, Wildish, *et al*. (2005) filtered out the sexual scripts of young people in Kenya through a series of focus group discussions, resulting in an in-depth understanding of how sexuality is experienced by young Kenyans and the socio-cultural contexts in which it is embedded. Other authors have studied aspects of adolescent sexuality, e.g. masculinity scripts or male perceptions on sexuality (Izugbara 2004, 2008), gender dynamics (O'Sullivan, Harrison, Morrell, Monroe-Wise & Kubeka 2006), the first sexual encounter (Izugbara 2001), multiple relationships (Izugbara & Modo 2007), or in a specific risk context such as funeral rituals (Njue, Voeten & Remes 2009), or on the way to school (Hampshire, Porter, Mashiri, Maponya & Dube 2011).

This study focuses on young people in Rwanda for two main reasons. First, there is very little information on sexual relationships of young Rwandans. While data are available on the basic sexual and public health outcomes (as age of sexual debut, number of sexual partners, condom use), to our knowledge, no studies have yet been published on Rwandan young people's thoughts, perceptions and experiences with sexuality and sexual relationships. Second, the data gathered in the study will be used to inform an intervention to reduce sexual risk in secondary schools.

### Objectives

This study aimed to gain an understanding of young Rwandans’ perceptions on sex and relationships, which is essential for formulating effective SRH promotion interventions. Through analysing the stories they spontaneously recount about sexuality and relationships, we assess their vulnerability for poor SRH and thereby formulate recommendations for interventions that more directly address the needs of young people.

We also aim to test the utility of a new qualitative technique: the mailbox technique. We aim to assess if information we get from this technique can be used to inform an SRH promotion intervention.

### Vulnerability: a theoretical framework

In analysing and interpreting the results, we use a framework developed by Delor and Hubert (2000) that makes the concepts of ‘risk’ and ‘vulnerability’ tangible and operational. We opted for this theoretical framework since the results of our study were to inform an intervention that aimed to reduce sexual risk. We used it as an interpretative framework.

Risk in our study is the risk of having poor SRH status (HIV, other sexually transmitted infections (STIs) or an unplanned pregnancy). Vulnerability focuses on why and how some individuals or groups are exposed to higher levels of risk in their lives than others. For example, individuals may have the same chance of getting HIV when having unprotected sex with an HIV-positive partner. However, the chance of having unprotected sex with an HIV-positive partner is higher for some individuals or groups, making them more vulnerable.

Delor and Hubert argue that this social aspect of vulnerability can be understood on three levels: first, the social trajectory: each individual goes through different phases in her/his life course, which generate different risks; second, the interaction: HIV infection requires two individuals/trajectories to meet. Individuals may adopt different risk-related behaviours according to their position or status in the interaction; and finally, the social context influences the moments, stakes and forms of encounters between different trajectories. Building on Watts and Bohle (1993), Delor and Hubert formulate a three-by-three matrix, by crossing these three levels with three further ones. Specifically, these are exposure (the risk of being exposed to a certain situation), capacity (possessing the necessary resources to cope with this situation) and potentiality (the risk of being subjected to serious consequences as a result of the situation).

## Methods

### Study setting

Six schools were selected with the aim of including a variety of schools encompassing: status (public/private), setting (urban/rural) and education level (lower/higher-secondary education). All schools included are mixed gender and have boarding facilities. Between 50% and 95% of students reside at the schools, which have separate dormitories and sanitary installations for boys and girls. One school had a particular religious affiliation (school 3: Muslim). In another, which was later excluded, the mailbox technique was not correctly implemented (Table 1).

**Table 1.** Participating schools.

School	Status	Boarding school or not	Setting	Education offered
School 1	Private	Boarding school	Urban	Lower- and higher-secondary education
School 2	Public	Boarding school	Urban	Lower-secondary education
School 3	Private	Boarding school	Rural	Higher-secondary education
School 4	Public	Boarding school, but large number of day students	Rural	Lower- and higher-secondary education
School 5	Public	Boarding school, but large number of day students	Semi-urban	Lower-secondary education
School 6 – excluded from the study	Public	Boarding school	Rural	Lower- and higher-secondary education

The secondary-education system in Rwanda is divided into two parts. In lower secondary, the first three years, students all follow the same programme. During higher-secondary education, the final three years, different study sections are offered. The age of the students in secondary education ranges from 12 to 30 years and even older. The median age of students in the last year of secondary education in the schools included in this study is 21 years (IQR of all students in the six schools = 17–20 years).

In each school, the Rwandan Government installed a mandatory ‘anti-AIDS club’. This club is tasked with motivating students to take preventive efforts against HIV infection. The students also receive information on biological aspects of SRH in biology classes.

### Procedure

Originally, we intended to use a diary method, in which young people could write about their experiences and thoughts concerning sexuality or relational issues over a certain period. However, as the majority of secondary-school pupils in Rwanda attend boarding schools where there are no safe spaces to store personal writings, privacy could not be guaranteed. Therefore, we sought a way in which young people could freely and voluntarily express their ideas on paper in a secure and private setting. The idea of a mailbox emerged, which offered the advantages of anonymity and spontaneity.

Six secondary schools were given a mailbox and asked to install it in a place with a large passage of students, away from signs of authority such as the principal's office. The mailboxes were locked and the keys kept by the principal investigator. Instructions were attached to the mailbox and the students received detailed information on the objectives of the study in a school assembly. It was emphasized that only the researchers would read the letters and that participation was voluntary.

The following instructions were placed on the mailbox (translated into the local language Kinyarwanda):

What's your story?

To better inform young people about relationships and sexuality, we must understand what they really think about these issues. What are your experiences with relationships and sexuality and what are your ideas, secrets, wishes, desires, fears … on these topics? In order to share your stories and ideas anonymously, we invite you to write them and then post them in this mailbox. We will collect the letters on [date specified].

We thank you for your story!

This message was followed by practical information, such as telling the students they could write in any language and on any topic concerning sexuality. The information was also circulated via a letter to the students.

Mailboxes were given to the six schools in March 2009. When returning to the schools three months later, we found that two schools had not installed the mailbox; in another school the lock had been stolen. The three other schools did hang the boxes, but on two of them the instructions had disappeared. We collected 25 letters, of which 14 letters had low relevance describing complaints about internal school issues, such as canteen food.

After analysing the remaining letters and discussing the poor results, it was decided to reattempt the study. In September 2009 all six schools were revisited. The researchers checked whether the mailboxes were installed in a correct place on the school grounds and reattached the instructions. Students were gathered and given repeated explanations. It was decided to leave the mailboxes on school grounds for a period of six months, including a three-month holiday period.

**Fig. 1.**
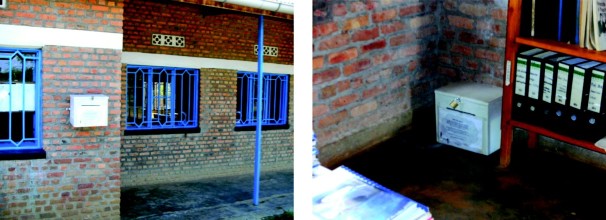
The left image shows a mail box that is correctly installed, the right image is an example of a mail box on the school library floor.

The schools were revisited in March 2010. In five schools, the mailboxes and instructions were still hanging upon our return. In one school, the lock was stolen for the second time. This school (school 6) was excluded from the study. One hundred and sixty-one letters were collected in the five remaining schools, bringing the total to 186 letters. One school (school 5) accounted for more than half of the letters (83 letters), with the remainder equally divided over the four other schools (Figure 1).

### Analysis

Almost all letters were written in Kinyarwanda. They were translated into English and analysed in QSR NVivo 9 (QSR International Pty Ltd., Melbourne, Australia). In a first step, the letters that did not deal with SRH or relationship issues were excluded from the analysis. Second, a closed coding system was applied, using the theoretical framework of Delor and Hubert (2000) as a guideline. We assessed the letters on their information on social trajectory, interaction, social context and their sub-categories exposure, capacity and potentiality. Within these categories, we developed grounded sub-categories. The coding was done twice by the same researcher with a four-month time lapse in between.

### Ethical approval

As part of a larger study on the effectiveness of a peer-education programme for HIV prevention, the mailbox study was approved by the Ethics Commission of the Ghent University Hospital (2008/485), the Rwandan National Ethics Committee (42-RNEC-2009), the Rwandan National AIDS Control Commission (130/2009/INSR) and the Rwandan Institute for Statistics (0135/CNLS/2009/S.E).

## Results

Of the 186 letters, 32, equally divided over the schools, were considered not relevant. They mostly contained complaints about school issues, teachers or the quality of the food served at school (*n* = 28). Four letters were directed to the Rwandan Red Cross, a partner in the study, with requests for support. The remaining 154 letters all contained information on topics related to SRH. Seventy-nine writers identified their sex: 42 were girls and 37 boys. The median age of those who provided their age (*n* = 15) was 17 (mean 17.9) with a range from 15 to 24 years.

### General tone of the letters

The sexual relationships described in the letters can be divided into two distinct groups. First, experimental sex, which takes place unprepared between two young people and is driven by sexual desire (*n* = 21). Due to their ad hoc nature, these sexual interactions are often unprotected. Second, transactional sex between a young girl/boy and an older man/woman after a process of negotiation (*n* = 40). This type of sexual interaction is particularly risky for HIV transmission, since older partners are more likely to be infected with HIV and other STIs, and are likely to have multiple partners.

One other type of relationship is described to a lesser extent: sex with someone in a superior function. For example, teachers having sex with students in exchange for marks (*n* = 3). Sex with soldiers is described by students who live near a military base (*n* = 5). These sexual relationships are mentioned, mostly in the third person, but are not elaborated upon further.

Students are targeted by soldiers, in fact pregnant girls who drop out of school most of time are made pregnant by them. (Girl, letter 75)

Relationships with emotional involvement, love or being in love were not described. Rather, it is described that ‘love’ can be used to take advantage of girls (*n* = 4).

It happens that a boy tells a girl that he loves her and starts conversing while touching her. He keeps telling her ‘I love you, let's sleep together’. If she is easy-going she agrees, while in reality the one that makes her pregnant does not care for her. (Girl, letter 47)

When writing in general and impersonal terms, the authors almost always describe sex in a negative way, as an act that is wrong and has severe consequences, referring to sexual intercourse as ‘sex(ual) delinquency’. This could be because in Rwanda the legal age of consent is 18 years, and the legal age of marriage is 21 years (Interpol 2006), while sex before marriage remains taboo. It seems that sex can only have negative consequences according to the writers: HIV/AIDS is considered a serious threat and unwanted pregnancy is a frequently mentioned outcome, resulting in exclusion from school. Hence, the general tone is that sexual intercourse is to be avoided by young people. On the other hand, when writing personal stories, the discourse of young people recognizes that they feel a desire to have sex. In these stories, sex is described as the result of a physical urge and a source of pleasure, and not necessarily as a negative act.

### Exposure: dominant types of sexual relationships

#### Experimental sexual relationships

In their letters, it is clear that young people are curious and experiment with sex, or wish to do so. Physically, they feel a need to start having sex (*n* = 21). The Rwandan students, especially those in boarding school, live in a very particular context. Most of them live in boarding schools with hundreds of other young people of both sexes without much adult supervision and only return home two or three times a school year. Even though many activities are organized in these boarding schools, the students still have a lot of free time. Experimental sex is also seen as a game, a way to pass time.

I haven't yet had sex, but if I take myself as an example I want to have sex, I want to experience it. (Boy, 19, letter 1–7)

I have boyfriends and there is nothing our love is based on other than making fun in pornographic games of all kinds. We don't have any plan to get married. (Girl, letter 110)

Girls are seen as provoking male sexual urges through the way they dress. This suggestion is only made by boys. The authors describe girls as seducers, not leaving boys another option than to have sex with them (*n* = 4).

I personally think that the origin of all of these things [HIV, STIs, pregnancy] is girls. When they put on clothes that show the navel and miniskirts, it drives a person to take her out!!! (Boy, letter 124)

This experimental sex mostly takes place unprepared. Sexual decisions are made when the opportunity emerges, often resulting in unprotected sex (*n* = 9).

When boys or we are involved in such bad acts [sex delinquency], the problem is that we do not remember that there is an incurable disease that is facing us, AIDS. [ … ] We prefer having fun ignoring that life is life. (Girl, letter 82)

As usual when a boy becomes a teenager he has the feeling of having sex. [ … ] Having done this, he feels such a desire of always doing it and most of the time he has unprotected sex. (Letter 83)

Experimental behaviour does not only occur with regard to sex. Authors also mention the use of alcohol and narcotics (*n* = 5), and their links with sex. However, substance abuse is always mentioned in the third person; none of the writers told a personal story on this topic.

There is another category of young people who rush to sex because of taking drugs. (Boy, letter 137)

In general, young people feel the need to belong to a group and are concerned about group loyalty. Since for most young people in our study the family lives far away, approval of their friends and peers is all the more important. As a consequence, young people may have sexual intercourse as a result of pressure from their peers (*n* = 5).

Young people in schools always want to please their friends. Wherever they are, one wants to please another. And it is because of this that they have sex. (Boy, letter 137)

There is a girl who asked me to sleep with her so that I give her 200 Francs [0.25 Euro]. I refused, but what makes me be sad is that she is telling everyone that I am a coward. (Boy, letter 21)

Another aspect that is commonly associated with growing up is feeling invincible and invulnerable. This was not found in the letters. Low risk perception is rare in the letters. If anything, most writers actually seem to overestimate their risk – stating that sexual intercourse almost automatically leads to both pregnancy and HIV infection.

#### Transactional sexual relationships

The second prototypical story is about a girl who is jealous of her peers' possessions and wants to obtain the same through sexual intercourse (*n* = 40). She looks for a boy or a man who can offer her the things she needs. The girl sees herself negotiating and working for these possessions. We could distinguish two types of transactional relationships: those needed for survival and those needed to obtain goods for peer acceptance. Even though many state that poverty is the main reason for HIV infection, stories about survival sex are rare.

I think AIDS is raging among teenagers because of their passion for possessions. (Girl, 15, letter 60)

For instance a girl may come to school with cheaper body lotion from her parents. Others may get expensive body lotion and noticing this she may become jealous and goes to find those who can offer her such body lotion. (Girl, 17, letter 1–6)

A girl is behaving like someone from a rich family while she was born in a poor family. She puts herself at a high class level. This drives her to sex delinquency which also leads to HIV infection. (Girl, letter 92)

It can happen that you are an orphan or poor and you go to look for a job. Then you find a widowed woman who wishes to have sex with you, so she starts showing you her wealth. (Girl, letter 71)

The initiative might also lie with the wealthy man/woman, offering a girl/boy money or possessions in order to make her/him have sex with him/her. In the sexual culture of Rwanda, accepting a gift equals agreeing to have sex with that person. This can be in the short term (after one gift) or over a longer period of time (after a series of gifts). Transactional sex is not necessarily a one-time thing. Long-lasting relationships can be built upon gift exchanges. It is not uncommon that the rich gift giver and the receiver have more than one girl-/boyfriend.

A boy comes and tells you that he is going to give you money to buy anything you want, and when you receive it, he immediately tells you that since he has helped you solve your problem, you have to solve his. (Letter 80)

For if she agrees that he buys her something she also agrees to do other things [have sex]. (Girl, letter 47)

Old men are very active in seducing students and other young children whom they tempt with money and telephones. They ask them to come when they need them. They spend nights together in hotels. They don't use a condom because they say that they don't get satisfied. (Girl, letter 140)

**Fig. 2.**
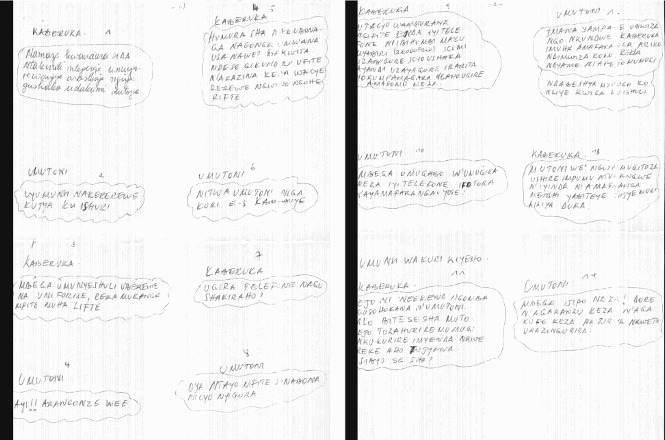
Part of a comic strip that warns of the dangers of older, rich men (‘sugar daddies’) seducing young girls (in Kinyarwanda); ‘I wrote this comic strip with the intention to talk about adults who tempt students and who are usually referred to as sugar daddies. Kaberuka was a rich man but had AIDS and was not faithful to his wife. One day he met with a student named Umutoni. He felt much love towards her and searched ways of tempting her into having sex with him. [ … ] Kaberuka tempted her until he made her pregnant and infected her with AIDS. In order to meet with Kaberuka, she was telling her parents that she was going to the weekend class program. [ … ] As for Kaberuka, he later on died of AIDS because he was not taking antiretroviral drugs and was spreading AIDS everywhere. Follow the passage as it is written on the following pages’ (Letter 72).

The power balance in such relationships is not necessarily in favour of the older/wealthy partner. On the contrary, often girls see themselves as possessing the main bargaining chip and working to obtain a certain good. They are the ones who decide on the price for their body (*n* = 7). This is of course not the case for survival sex, in which the rich partner is dominant (Figure 2).

There are merchants who have a lot of money and who beg me to have sex with them in exchange for their money. (Girl, 18, letter 1–10)

Then the girl says ‘I can't live without a telephone, that is stupidity. I must sell my body.’ The beginner tries to hide but after some days she does it openly. [ … ] One girl works for a telephone, another one says I am going to work for airtime, another one says me I am going to work for body lotion. (Girl, 18, letter 18)

### Capacity: coping with the risk

Ideally, young people would possess personal resources that would protect them from HIV/STI infection or unwanted pregnancies. Personal resources can be knowledge of transmission and protection modes and access to SRH services and social support. However, the stories in the letters indicate a limited capacity by young people in dealing with their vulnerability. The stories indicate that information on SRH comes predominately from dubious or unreliable sources such as pornography or from peers. The letters show limited knowledge on topics of SRH. This can be derived from the questions they ask or the incorrect statements they make. Forty-nine authors asked a total of 114 questions. Topics that regularly return are the menstrual cycle for girls (*n* = 20) and the origin of HIV/AIDS (*n* = 7). Seemingly, the adults surrounding those young people, their parents and teachers, fail to inform them on this topic (*n* = 9).

You find that many [young people] get pregnant unprepared because nobody trained them on matters relating to their bodies. (Boy, letter 137)

Parents who refuse to tell their children the ways of AIDS transmission by saying that they are ashamed, they are doing them wrong. The child will become an adult without knowing anything. (Girl, letter 43)

In Rwanda, condoms are freely available in health centres, but young people never mention this service. Plenty of letters contain the suggestion that condoms should be more accessible and even distributed in the school free of charge (*n* = 20). This could mean they do not know they can obtain condoms from these facilities or that they have difficulties in accessing health centres. Those who have experience with using condoms associate it with reduced sexual pleasure.

I suggest putting in place a mechanism in secondary schools through which condoms can easily be accessed, especially since in boarding schools, sex scenes are frequent. (Letter 72)

I have now resorted to condom use and it doesn't feel well (sexual pleasure). I suffer a lot during such an act. (Letter 2)

### Potentiality: consequences of the risk

The consequences of risky sexual behaviour for the individual are considered severe and immediate. The writers mainly mention unwanted pregnancy and HIV/STI infection. Experience from the field teaches us that prevention messages in Rwanda often sensitize young people by telling the story of a young girl who is seduced by an unknown boy – a process likely to include alcohol and/or gifts – and after one sexual intercourse the girl finds herself pregnant and HIV infected.

Other consequences of a social nature mentioned are that HIV-positive or pregnant students are forced to leave school (*n* = 5) and that loosing virginity reduces a girl's value (*n* = 3). Not having sex, on the other hand, can lead to worries about future sexual performances and loss of face for boys (*n* = 3).

The first time I had sex I was 20. I got pregnant and had to give up my studies for three years. (Girl, 24, letter 3)

They [young people] have bad and different information on sex before marriage stating that when you get married you fail to perform sexual acts [if you did not have sex before marriage]. (Boy, letter 137)

Only one writer described the consequences of HIV infection on a hypothesized relationship stating that he would try to infect others so that he would not be alone. In general, writers state that HIV-positive persons should not be stigmatized, but also that they should avoid infecting others by not having sex. Another author sees negative consequences for society as a whole.

When I would learn that my beloved one is infected, I would immediately leave her and find another one. But if I find out that it is me who is infected, I would do my best to sleep with her and infect her. This would push me to start with her. All the girls who would like, I would have sex with, so that we can go together to Nyamata [district capital] to get antiretroviral drugs. (Boy, 20, letter 1–12)

This causes population growth and poverty. Young people give birth at early age and unexpectedly and some get AIDS infection, which results in orphans and children of the street. (Letter 83)

### Prevention programmes

Many students offered their thoughts about their preferred SRH promotion interventions. Fifty-eight requests for training on SRH were made. This additional training should focus mainly on biological aspects of SRH, such as physical health, and HIV/STIs. Also, advice on how to avoid temptations is needed. Students prefer an external expert to provide regular training on these topics, while also indicating that parents should inform their children. In addition, media (radio and movies) are suggested as an interesting information tool. Teachers are not identified as a preferred information source.

As for the content of prevention messages, young people put great emphasis on abstinence (*n* = 29). They consider condom use a second and less preferable option, only to be used in the case one fails to abstain. Nevertheless, many young people plea for free distribution of condoms in the schools (*n* = 20; Table 2).

**Table 2.** Comprehensive overview of a young person's vulnerability to poor SRH during adolescence in a school context in Rwanda.

	*Social trajectory* – puberty/adolescence	*Interaction* – experimental and transactional sexual intercourse	*Social context* – boarding schools in a resource-poor setting
*Exposure* – the risk of being exposed to a certain situation (i.e. unprotected sex)	• Sexual experimenting – urge to have sexual intercourse	• Age difference: older partners have more risk of having HIV/STI	• HIV/STI prevalence in society
	• Alcohol/substance abuse – less control over sexual decision-making		• Setting: boarding schools
	• Peer pressure for being part of a group:		
	(a) Of those sexually active		
	(b) Of those possessing nice goods		
*Capacity* – disposing of the necessary resources to cope with this risk	• Limited knowledge on SRH	• Negotiating power: older/rich partners versus girls working for goods	• Poverty
	• Limited support of important adults	• Power imbalance: teachers/soldiers/merchants as partner	• Legal age of consent
	• Difficult access to condoms and health services	• Gender: girls as seducers and boys as not having control over their behaviour	• Taboo on youth sexuality and emphasis on abstinence
*Potentiality* – the risk of being subjected to serious consequences as a result of the situation	• HIV/STI infection	• Leaving HIV infected/pregnant partner	• Population growth
	• Pregnancy	• Trying to infect partner	• Poverty
	• Exclusion from school		
	• Exclusion from peers:		
	(a) Reputation damage		
	(b) Not possessing nice goods		

Note: Based on the vulnerability framework of Delor and Hubert (2000).

All of us young people must abstain completely. Those who fail to abstain can use a condom. (Girl, 15, letter 60)

I would like you to bring us condoms because they are very much needed here at school. (Letter 107)

Other strategies include more restrictive rules and laws (*n* = 7), HIV testing (*n* = 11) and empowerment (*n* = 2).

Tightening security so that young people know that if they are caught [having sex] they are punished appropriately. (Boy, letter 42)

I, personally, ask you to send doctors to our school each month to have us tested. (Letter 70)

I think we must know to refuse or to accept. If a boy asks you for sex and you accept you don't have to blame him when you face consequences. If you refuse, you show him that you don't joke. (Girl, letter 136)

## Discussion

### Vulnerability of young people: social trajectory, social context and interaction

People are vulnerable to poor SRH because of the types of sexual relationships (interaction) they engage in. This engagement is influenced by young peoples' stage in life (social trajectory) and the environment (social context) in which it takes place.

Young people take up a specific place on the social trajectory. They go through a period of physical and emotional maturation on their way to adulthood, constructing their adult identity by exploring and experimenting, including sexual experimentation. Furthermore, peer pressure is common in this life phase, and learners report pressure to have sex and to have the right possessions. Furthermore, this turbulent period seems to generate some seemingly contradictory opinions on sex, relationships and preferred prevention methods: experimental, irrational and ad hoc sex co-exists with planned, rational and negotiated transactional sex; the physical desire to have sex for pleasure is at odds with the description of ‘sexual delinquency’; the emphasis on abstinence seems to contradict the requests for free condom distribution in the schools. Young people's limited knowledge and experience makes them vulnerable to peer pressure and unhealthy decisions. However, as we only know the exact position on the social trajectory for some of the respondents, we cannot draw general conclusions on this item.

This particular vulnerability is enhanced by the proximate and distal context in which this maturation process takes place. The situation in which the study participants live – boarding schools with relatively limited supervision – allows for much more contact between boys and girls, but few opportunities for planned sexual intercourse. The letters also identified several other contextual factors that influence sexual decision-making: social norms, gender imbalance and economic factors. First, sex between young people is taboo and considered morally and legally (under 18 years) wrong. Virginity at marriage is still considered the norm for girls (Musabyimana 2006). This makes it difficult for young people to understand and express the positive aspects of sex. Second, girls are seen as the provokers of sexual desire in boys both for experimental and for transactional sex, while boys seemingly only act upon their physical needs. This corresponds with a study on sexual relationships among young people in developing countries by Brown, Jejeebhoy, Shah & Yount (2001) in which it appears that young people encourage premarital sexual relationships for males, but not for females. Subsequently, it appears respondents are able to justify the fact that girls carry the largest consequences for inappropriate sexual behaviour, e.g. being punished for becoming pregnant. Third, economic reasons may influence sexual decisions. Young people have sex in exchange for money and goods. Survival sex is rare among responses, but relative disadvantage does influence sexual decisions, in the sense that young people need money to buy less essential goods, such as telephones and body lotion that others may have. Remes, Renju, Nyalali, Medard, Kimaryo, Changalucha, *et al*. (2010) call this ‘the desire to lead a modern life’, indicating that girls are ‘active agents’ and not merely ‘vulnerable victims’ (Hunter 2002; Silberschmidt & Rasch 2001; Wamoyi, Fenwick, Urassa, Zaba & Stones 2010; Wamoyi, Wight, Plummer, Mshana & Ross 2010). The less urgent need for these products in comparison to survival sex surely might put young people in a stronger negotiating position. However, they seem to use this power to negotiate more goods, instead of safe sex.

At this life stage and social context, young people engage in sexual interactions, mainly experimental and transactional sex. They presently lack the necessary information, support and resources to make healthy decisions. It is clear from the letters that these young people have a hunger for information on sexuality and reproductive health. A large number of letters requested trainings on SRH and many students had written down questions. This might indicate that they feel they do not have trustworthy persons in their direct environment that can answer these questions in confidence. The large number of questions relating to biology (e.g. on the menstrual cycle) indicate that, even though SRH is in the school curricula, factual information is not sufficiently and timeously disseminated.

None requested an intervention to alter the social context. It is not clear if the youth are fully aware of the importance and their ability to alter the social and contextual factors they identify in their letters on their sexual decisions. For example, gender inequality – girls are seen as provokers of sexual desire – is recognized as a fact or a permanent feature of their environment, but not as a factor amenable to change through female empowerment.

Finally, we must also be aware of what remains unwritten from the letters. A positive discourse on adolescent sexuality and relationships is almost absent. While young Rwandans do have sex for pleasure, the values attributed to adolescent health are negative and sinful (‘sex delinquency’), and abstinence is considered the best behaviour. This partially corresponds with findings from Harrison, who found two competing discourses in South African youth – love and romance versus stigma and secrecy – and young people are largely left to themselves in making sense of these competing discourses (Harrison 2008). Also, Rwandan youth lack the social support and information to make healthy and informed distinctions between competing areas.

As the data were collected in secondary schools and the net enrolment rate in Rwanda is low (15%), they are not representative of the general adolescent population in Rwanda.

### Recommendations for SRH promotion interventions

The vulnerability of young people is determined by a complex interrelated web of mutually reinforcing factors. These factors carry different weights for different individuals and groups and in different situations. An intervention that takes into account these aspects is more likely to succeed than an intervention that targets just one or several aspects.

As the letters demonstrate that young people who are sexually active still have many questions on sexuality issues and safe sexual behaviour, we would recommend that SRH promotion interventions target younger children with key messages on sexuality and relationships, before the complex physical and emotional process of sexual identity construction starts.

Next to prevention messages for larger groups, the vulnerability of young people – prone to peer pressure and doubt – calls for an individual approach. Young people have many questions, concerns and uncertainties about sexuality. The creation of a ‘safe haven’ for them to be able to ask these questions and express their concerns is essential. Such a safe haven could take many forms depending on the situation. For our study population, this safe haven could be a mailbox where the students could post their letters and answers would be provided in writing, a website with the same purpose or an external expert coming to see the young people on a regular basis.

### Experience with the ‘mailbox technique’: limitations and opportunities

Almost half of the relevant letters came from one (semi-urban, lower secondary) school, even though all schools were given similar levels of information about the project. School administration and teachers were asked if they had undertaken actions to motivate their students to write the letters, which was not the case. The reason for this difference between schools remains unclear.

In one school, the lock was stolen twice. Upon inquiry, it was said that the lock was already missing from the first day, so it is unlikely that any letters were posted. Nevertheless, personal information may have been discovered by others (even without identity information). A more secure way should be found to ensure that letters are only seen by the research team. Emptying the mailboxes more regularly might also be important.

The mailbox technique took place in schools where a larger study on the effectiveness of peer education as a tool for HIV prevention and SRH promotion of youth was planned. Even though the study was initiated before the start of the intervention, the baseline survey already took place and it might possibly have influenced the content of the letters.

The study did generate a large number of stories related to sexuality of young people. However, the majority of the letters described young people in general and did not always include personal stories. This might be solved by asking a more direct, personal question or asking respondents to describe a recent personal experience. A less threatening way may be to propose a limited number of themes they can write about. If describing personal experiences is too sensitive, another option would be to ask them to write down recent conversations between peers on the topic (Swidler & Watkins 2007; Watkins & Swidler 2009).

There is no control over the selection of the participants. Therefore, this technique is preferably done in a relatively homogenous group. Participation bias, e.g. that older and more extrovert students are more likely to write, cannot be excluded.

Nevertheless, we believe the mailbox method to be a valuable tool for qualitative research. It allows participants to decide for themselves if they want to take part in the study (voluntary). They can participate when they want, over a long period in time. The method should assure that no co-participant, or in the case of our study, no teachers or fellow student, can access the information (confidential). The participants decide for themselves if they want to make themselves known (anonymous). And, most importantly, the participants are in charge of the topic discussed, allowing the researchers to find out what is really on their minds (participant-initiated). Furthermore, the method is low cost. An advised adaptation would be a shorter time span to write the letters (for example, over a month with weekly collection of letters).

## Conclusion

We can make conclusions on four major points. First, current efforts are not sufficient for young people. They still have major gaps in knowledge about sex. These gaps are not addressed in the existing initiatives (anti-AIDS clubs and biology classes). Furthermore, even though condoms should be freely available in health centres, young people still report a great need for condoms and many recommend distribution in the school. Second, interventions should focus on the two main sexual interactions respondents described, namely experimental and transactional sex. Third, risky sexual decision-making is influenced by many factors – the social trajectory, the social context and the interaction – and therefore SRH promotion interventions that are not related to all three aspects may be less effective. Finally, the mailbox method appears to be a cost-effective means of collecting information on young people's thoughts on sexuality and prevention methods.
